# A case report on transitory histamine intolerance from strawberry intake in a 15 month old child with acute gastroenteritis

**DOI:** 10.1186/2045-7022-5-S3-P61

**Published:** 2015-03-30

**Authors:** Alkerta Ibranji, Elida Nikolla, Gjustina Loloci, Ervin Mingomataj

**Affiliations:** 1 “Mother Theresa” School of Medicine, Tirana, Albania; 2 Regional Hospital of Saranda, Saranda, Albania

## Background

Histamine is a vasoactive amine found in different foods. Di-amine oxidase (DAO) released from apical cells of intestinal mucosa is the responsible metabolizing enzyme for ingested histamine. Histamine excess because of DAO reduced or inhibited activity may mimic an allergic symptomatology, often misleading to food allergy diagnosis instead of histamine intolerance. Viruses, as the main cause of acute gastroenteritis in children, induce apical cell death, lower DAO production levels and increase the intestinal permeability owing to direct and indirect virus -host interactions.

## Methods

A 15 month old boy patient presented in the hospital of Saranda with dyspnea, facial angioedema and larynx edema(voice hoarsening), generalized cutaneous symptoms, abdominal cramps/bloating, and diarrhea without cardiorespiratory alterations, 40 min after the ingestion of 100 ml yogurt with fresh strawberry. His mom reported an earlier (1 day before) mild episode with urticarial elements and pruritus only, localized on facial and abdominal area after banana yogurt ingestion that resolved spontaneously. Plain yogurt, plum, cherry and pear yogurt intake in previous weeks were well tolerated from the child. The patient had diarrhea and 38.5 0C fever for 4 days and was diagnosed with acute gastroenteritis. He was symptoms free 3 days before the allergy onset. Three days treatment with 1.25 mg/day desloratadine was prescribed for this patient.

## Results

Blood count, urine and fecal examination ruled out bacterial and parasite infections. In vivo and in vitro allergy tests applied 6 weeks after the allergic episode resulted negative. Mastocytosis and Eosinophilic disorders were ruled out from two consecutive urine 1- methyl histamine tests after the first one. The child tolerated the provocation test with strawberry and yogurt.

## Conclusion

Viral intestinal infections can be triggers of food allergy-like symptoms, especially in children. Low histamine containing foods may be a safer diet for children with acute gastrointestinal viral infections. A careful clinical history is a key element in differential diagnosis.

## Consent

Written informed consent was obtained from the patient’s parent or guardian for publication of this abstract and any accompanying images. A copy of the written consent is available for review by the Editor of this journal.

